# History, status and genetic characteristics of native cattle breeds from the Republic of Kazakhstan

**DOI:** 10.18699/vjgb-24-47

**Published:** 2024-07

**Authors:** A.K. Khamzina, A.A. Yurchenko, N.S. Yudin, P.Sh. Ibragimov, Y.S. Ussenbekov, D.M. Larkin

**Affiliations:** Kazakh National Agrarian Research University, Almaty, Kazakhstan; Institute of Cytology and Genetics of the Siberian Branch of the Russian Academy of Sciences, Novosibirsk, Russia; Institute of Cytology and Genetics of the Siberian Branch of the Russian Academy of Sciences, Novosibirsk, Russia; Kazakh National Agrarian Research University, Almaty, Kazakhstan; Kazakh National Agrarian Research University, Almaty, Kazakhstan; Royal Veterinary College, University of London, London, United Kingdom

**Keywords:** cattle, breeds, history, Kazakhstan, genetic characteristics, single nucleotide polymorphism, крупный рогатый скот, породы, история, Казахстан, генетическая характеристика, однонуклеотидный полиморфизм

## Abstract

This work provides a comprehensive review of the history, status, and genetic characteristics of cattle breeds in Kazakhstan. The current breeding status is analysed, including information on popular breeds such as Kazakh white-headed, Auliekol, Alatau, Aulieata, and Kalmyk, their production and economic significance. An overview of genetic studies using DNA fingerprinting, microsatellites, and SNPs aimed at identifying unique characteristics, genetic diversity, and genes under selection, as well as markers of economically important and productive traits of Kazakh cattle breeds, is also provided. The study examined the genetic structure of the Kazakh white-headed and Alatau breeds based on whole-genome SNP genotyping. Unique genetic components characterizing Kazakhstan cattle breeds were described, and comparisons were made with genetic data from other breeds. Structural analysis showed that the Kazakh white-headed breed contains genetic components of the Hereford, Kalmyk, and Altai cattle. The Alatau breed has a composite structure, containing components of the Brown Swiss, Braunvieh, Kalmyk, and Holstein breeds. The results not only reveal the genetic diversity and characteristics of cattle breeds in Kazakhstan and the historical development and current state of animal husbandry in the country, but also emphasize the importance of further research to identify adaptive and unique genetic markers affecting economically important traits of local breeds.

## Abstract (ru)

В этой работе мы представляем обзор истории происхождения, текущего состояния и генетических особенностей пород крупного рогатого скота (КРС) Казахстана. Отражена информация о современном состоянии разведения, в том числе о пяти наиболее популярных локальных породах – казахской белоголовой, аулиекольской, алатауской, аулиеатинской и калмыцкой, в рамках их производственной и экономической значимости. Приведен обзор генетических исследований по этим породам, направленных на выявление их уникальных характеристик, генетического разнообразия и генов, находящихся под давлением отбора, а также ДНК-маркеров экономически важных и продуктивных признаков казахстанских пород КРС. Представлены также оригинальные данные по особенностям генетической структуры казахской белоголовой и алатауской пород на основе результатов полногеномного генотипирования однонуклеотидных полиморфизмов. Описаны уникальные генетические компоненты, характеризующие эти породы КРС Казахстана, и проведено их сравнение с генетической структурой популяций этих же и других пород из Российской Федерации. Структурный анализ показал, что казахская белоголовая порода содержит генетические компоненты герефордской и калмыцкой пород, а также алтайского скота. Алатауская порода имеет композитную структуру и содержит компоненты бурой швицкой, браунви, калмыцкой и голштинской пород. Результаты демонстрируют генетическое разнообразие и особенности пород КРС Казахстана, развитие и текущее состояние скотоводства в стране, а также подчеркивают необходимость более детальных исследований для выявления генетических маркеров адаптации и экономически важных признаков местных пород.

## Introduction

For over 10,000 years, cattle have been an important element of agriculture and food production (Argynbaev, 1969; Dakhshleyger, 1980). The first mention of cattle breeding on the territory of Kazakhstan dates back to the Botai culture of the Bronze Age (III–II centuries BC). The study of bone remains indicates that these herds included mainly horses, but the remains of small and large livestock were also found (Adilova, Ilyassov, 2018). In the subsequent period (from the 15th to the 17th centuries), cattle breeding in limited quantities was noted in the Kazakh Khanate (Ratchnevsky, 1993; Allsen, 2001). Before the second half of the 19th century and early 20th century, Kazakhs practised, for the most part, a nomadic form of agriculture (Frizen, 2022). Nomadic cattle farming was extensive, using vast grazing areas rather than intensive farming methods in a limited area. They used pastures, where livestock lived throughout the year or almost all year round on natural pasture. This also determined the composition of the herd, which could only include animals able to pasture during the winter (Diarov, 1963; Argynbayev, 1969). To the most extent, these were horses and sheep, which made up the majority of the nomadic herd (Diarov, 1963; Tolybekov, 1971). In the 19th century, due to socio-economic changes, new forms of economy began to appear, such as semi-nomadic cattle breeding and agriculture.

A distinctive feature of semi-nomadic cattle breeding was that it was combined with agriculture (Tolybekov, 1971). Haymaking and farming are associated with an increase in the share of cattle in the Kazakh nomadic economy. Cattle became the main traction force in this type of farming. Another circumstance that contributed to cattle breeding in Kazakhstan was the emergence of a market for the sale of meat (Tolybekov, 1971).

Kazakhstan, a vast Central Asian country known for its diverse landscapes from steppes to mountains, has developed cattle breeds that meet specific human needs and are adapted to the local environment. Kazakh cattle breeds were formed in such a way as to live in the harsh, often extreme conditions of this country, while at the same time having high productivity (Diarov, 1963).

Modern local cattle breeds are characterized by their adaptability, sustainability, and ability to provide the population with necessary resources, such as meat, milk and hides (Kazkenova, Ainakanova, 2016). These breeds are the most important asset of Kazakhstan and the whole world. Therefore, it is necessary to study their unique genetics for subsequent improvement, as well as to create new commercial breeds that can maintain their outstanding properties in the harsh, sharply continental steppe climate of Kazakhstan and other countries.

In this work, we will review the literature describing modern cattle breeds in Kazakhstan, their commercial properties and genetic characteristics, and also present our data on the genetic structure of populations of two breeds: the Kazakh white-headed and Alatau based on data from whole-genome genotyping of samples of these breeds from Kazakhstan and the Russian Federation and their comparisons with other breeds

## Current status, distribution area and description of breeds

**Fig. 1.**
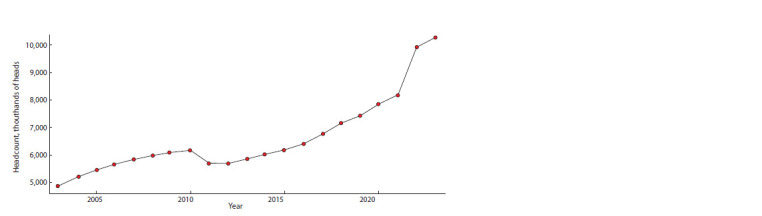
Total headcount of cattle in the Republic of Kazakhstan from 2003 to 2023 (www.stat.gov.kz).

According to the Bureau of National Statistics of the Republic of Kazakhstan, as of March 1, 2023, the total number of livestock in the region is over 10 million heads (Fig. 1). This figure is higher than in previous years and indicates a positive growth trend of ~4 % per year (www.stat.gov.kz).

There are 23 cattle breeds in the country, registered in the information and analytical system of Kazakhstan (www.plem. kz), including four breeds that resulted from crossing imported breeds with local livestock and are well adapted to the harsh climatic conditions of the region (Alatau, Aulieata, Kazakh white-headed and Auliekol) (Diarov, 1963; Torekhanov et al., 2006). These breeds have unique features, such as adaptation to harsh climatic conditions (extreme temperatures) and limited access to feed (Torekhanov et al., 2011), resistance to local diseases and parasites (Sattarova et al., 2023), high meat and dairy productivity in the country’s conditions (Torekhanov et al., 2011). To create these breeds, breeds imported to Kazakhstan from other countries were used to improve the economic characteristics of local livestock or adapt to new conditions of keeping and growing (Kazhgaliyev et al., 2016; Zhumanov, Baimukanov, 2020; Ulimbashev et al., 2023).

The main imported breeds that are now successfully bred in Kazakhstan include Kalmyk, Angus, Hereford, Holstein, Kholmogory, Limousin, Santa Gertrudis and others. According to the Republican Chamber of Dairy and Combined Cattle Breeds of Kazakhstan (www.qazaqsut.kz), which includes Alatau, Aulieata, Holstein, Black pied and other breeds of this productivity, the number of stud farms for 2023 is 628 farms, and the number of commercial ones is 1,271 farms. Most dairy and combined productivity cattle are Simmental and Holstein breeds

**Fig. 2.**
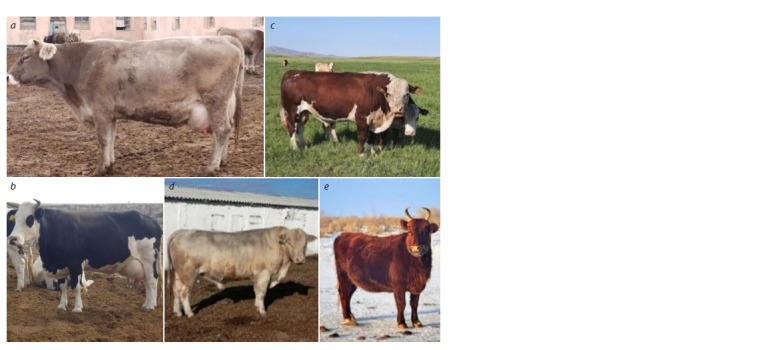
Alatau breed (a); Aulieata breed (b); Kazakh white-headed breed (c); Auliekol breed (d); Kalmyk breed (e).

Alatau cattle breed – meat and dairy productivity breed (Fig. 2a). Research on breeding the Alatau breed was carried out in 1930–1950 in the Kirghiz SSR and the southern regions of the Kazakh SSR by crossing local cattle with animals of the Kostroma and Brown Swiss breeds (Nysanbaev, 2004). The breed is adapted to living in high mountain areas, its colour is mostly brown, of different shades. As of the beginning of 2024, the population of breeding cattle of this breed is about 7 thousand heads, which is ~2.8 percent higher than the previous year (www.qazaqsut.kz). This breed is mainly bred in the Almaty and Turkestan regions of the Republic (www.gov.kz).

The Alatau cattle breed from Kazakhstan has been the subject of several studies aimed at improving its breeding and rearing. Thus, A.D. Baimukanov and co-authors (Baimukanov et al., 2021) focused on the effective breeding of the Kazakh population, while S.K. Abugaliev and co-authors (Abugaliev et al., 2020) studied the growth and development of heifer calves under various rearing technologies

The Aulieata dairy production breed (Fig. 2b) was bred in the Kyrgyz and Kazakh SSR by crossing local cattle with Dutch cattle and subsequent inbreeding of crossbreeds (Nysanbaev, 2004). It was first tested in 1952. A distinctive feature of the breed is its adaptability to breeding in hot climates and resistance to blood-parasitic diseases. The colour of the animals is predominantly black-and-white, but light grey is also found. The Aulieata breed fattens well. The animals are characterized by a purely milky body type, a well-developed udder, and correctly positioned limbs (Nysanbaev, 2004). As of 2024, the number of pedigree cattle of the Aulieata breed in Kazakhstan is about 1 thousand heads (www.qazaqsut.kz), with the main breeding happening in the south of the country (www.gov.kz).

The Kazakh white-headed meat production breed was developed in the USSR in 1930–1940 (Fig. 2c), and it was officially tested in 1950. The selection was carried out by complex reproductive crossing of a breeding stock of local Kazakh and partly Kalmyk cattle with Hereford bulls, as a result of which the cattle acquired the best qualities of all these animals: high adaptive ability, strong constitution, early maturity and high meat yield (Porter, 2016). The colour is red, of varying intensity, with a white head, chest, belly, lower limbs and tail brush. There are animals with white markings on the withers and rump; the front part is better developed than the back part; the hair is thick and short in summer, and long and slightly curly in winter (Dmitriev, Ernst, 1989; Nysanbaev, 2004). The total number of pedigree cattle of the Kazakh white-headed breed in 2022 is about 500 thousand heads, including about 200 thousand cows (www.gov.kz). The breed is bred countrywide, but the largest population is in the East Kazakhstan region. The Kazakh white-headed cattle breed makes a significant contribution to beef production in Kazakhstan (Bozymov, 2018).

The Auliekol breed was created by a complex reproductive crossing of three specialized meat breeds: Kazakh whiteheaded, Charolais and Aberdeen Angus (Fig. 2d ). Per the international classification, the breed belongs to large breeds of beef cattle. It was registered in 1992 (Nysanbaev, 2004). The breed is located mainly in the Kostanay region; it was also imported to the farms of the Pavlodar, North Kazakhstan, Almaty, and Karaganda regions. As of 2022, the number of pedigree cattle of the Auliekol breed is about 70 thousand heads, of which approximately 33 thousand are cows (www. gov.kz). The specialized Auliekol meat breed is characterized by good early maturity, high yield and quality of meat, high growth energy, and adaptability to local conditions. The colour of the animals is light grey, 70 % of the livestock are polled. Animals have a strong constitution. In winter, they grow thick hair and are well adapted to harsh natural and climatic conditions of a sharply continental climate (Nysanbaev, 2004). In summer, animals quickly gain weight, easily tolerate heat, and in winter they are tolerant to frost when outdoors.

The Kalmyk meat production breed has been bred in Kazakhstan since the 17th century (Fig. 2e). It was introduced by nomadic Kalmyk tribes more than 350 years ago from the western part of Mongolia and China (Bichurin, 1991; www. qalmaq.kz). The final formation of the Kalmyk breed took place in the conditions of a nomadic economy with yearround grazing of animals. Cows of the Kalmyk breed are, in general, medium in size and compact in build (Narmaev, 1963). The colour of the animals is red, with white markings on the head, belly or limbs. In winter, cows of the Kalmyk breed grow thick hair. As of 2022, the total number of breeding cattle of the Kalmyk breed is about 23 thousand heads, including about 15 thousand cows. The Kalmyk breed is mainly bred in the Zhambyl and Turkestan regions of Kazakhstan (www.gov.kz).

The Kalmyk cattle breed, which belongs to the group of Turano-Mongolian breeds (Yurchenko et al., 2018a), has high adaptive abilities and similar production and reproductive characteristics to the Mongolian breed (Fedotova et al., 2020). The productivity of Kalmyk bulls varies depending on breeding methods, while bulls of the Kalmyk breed of Buryat selection have a higher live weight compared to bulls of Kalmyk and Rostov selections (Lumbunov, Garmaev, 2021).

## Genetic characteristics of cattle breeds in Kazakhstan

Molecular genetic studies of Kazakhstan cattle breeds have so far been carried out using DNA fingerprinting, microsatellites, and SNP markers. These DNA markers are highly informative and variable for studying genetic diversity. However, in most cases, the analysis includes a limited number of markers, which does not provide a comprehensive study of the animal genome

Population structure. Analysis of the genotypes of three cattle breeds in Kazakhstan (Terletsky et al., 2019), Alatau, Kazakh white-headed and Auliekol, was carried out by DNA fingerprinting using DNA probes, which revealed the highest degree of genetic similarity in animals of the Auliekol breed (BS = 0.64), then in the Alatau breed (BS = 0.54), and the smallest, in the Kazakh white-headed breed (BS = 0.51). The genetic distance between the Kazakh white-headed and Auliekol breeds was the smallest (D = 0.025), which confirms their known genetic relationship. The Alatau breed showed the highest distance from the Kazakh white-headed and Auliekol breeds (D = 0.055 and D = 0.060, respectively). Heterozygosity (H) values are higher in the Kazakh white-headed breed (0.54), which exceeds the value of the Auliekol breed (0.38), confirming the higher genetic variability of the former breed (Terletsky et al., 2019).

Analysis of 12 microsatellite loci confirmed the relationship of the Kazakh white-headed breed with the Hereford breed, which is associated with the use of Hereford bulls for its creation (Shamshidin et al., 2019; Abdelmanova et al., 2021). This is confirmed by data from genome-wide genotyping of 154 thousand SNP markers, where animals of the Kazakh white-headed breed of Russian selection formed a cluster both in principal component analysis (PCA) and in structural and phylogenetic analyses, with the Hereford breed (Yurchenko et al., 2018b; Yudin, Larkin, 2019; Beishova et al., 2022a). On the other hand, the Kazakh white-headed breed has a high level of genetic diversity and has retained a significant fraction of Turano-Mongolian genetic components, which most likely originate from local Kazakh cattle and Kalmyk breeds.

Clustering of SNP markers revealed the genetic relationship of the Alatau breed with the Kostroma, Brown Swiss and Braunvieh breeds, which confirms the known history of the formation of the Alatau and Kostroma breeds (Yudin, Larkin 2019). Of the microsatellite alleles found in museum Kalmyk cattle samples, more than 80 % were also present in modern representatives of the breed (Abdelmanova et al., 2021).

As a result of genome-wide genotyping of SNP markers, a genetic relationship was revealed between the Kalmyk breed and the Serbian Busha breed (Iso-Touru et al., 2016). In turn, the Auliekol breed showed heterogeneity using SNP genotyping of 154 thousand markers, forming its own cluster in PCA and structural (ADMIXTURE) analyses (Beishova et al., 2022a).

The distribution and frequency of regions of homozygosity (ROH) in the genomes of the Kazakh white-headed and Auliekol cattle breeds were studied as well (Beishova et al., 2022b). In this study, it was shown that the Kazakh whiteheaded breed had a higher number of ROHs (55.976) compared to the Auliekol breed (13.137). Calculation of the average ROH length showed differences between the values of the Kazakh white-headed (211.59 ± 92.98 Mb) and Auliekol (99.62 ± 46.48 Mb) breeds.

Genes under selective pressure. When analysing genetic signatures of selection in the Kazakh white-headed breed, regions of the KIT, KITLG and EDN3 genes were identified, associated with white, roan coat colour and the “white head” phenotype, respectively (Yudin, Larkin, 2019). Analysis of haplotype frequencies from genome-wide genotyping data showed that the Kazakh white-headed breed exhibits signals on chromosome 6, in the LCORL-NCAPG gene region, which has been associated with a number of growth traits in cattle (average daily weight gain, muscle development, and carcass traits). The selection was also found in the interval on chromosome 14 containing the DGAT1 gene, which contributes to milk fat content.

The FKBP2 gene, which has been associated with milk protein yield and content, was found to be under selection in the Kazakh white-headed breed. In the Kalmyk breed, the areas under selection were in the region of the HMGA2 gene, which is associated with growth in cattle, and the TRPV5 gene, associated with hypocalcemia and postpartum paresis in cattle (Yurchenko et al., 2018b). In the Kalmyk breed, as well as in other Russian breeds, it was found that the RAD52 gene was subject to selection pressure. This gene is associated with DNA repair and is involved in antiviral defence processes (Yudin, Larkin, 2019).

Genetic markers of economically important traits. Analysis of the association of genotypes for the calpain (CAPN1) and somatotropic hormone (GH ) genes with productivity traits showed that Kazakh white-headed animals homozygous for the CAPN1 (CC) locus and homozygous for the GH (VV) locus are significantly superior to animals without the C and V alleles based on such characteristics as milk productivity, average daily body weight gain, pre-slaughter body weight, slaughter weight, carcass weight, pulp weight, chemical composition and histological characteristics of meat (Plakhtukova et al., 2020). Genetic markers such as blood group antigens A1, A2, D’, W, V, and Z have been identified in the Kalmyk breed, which may have potential implications for selection and breeding (Chimidova et al., 2022).

A study of cows of the Aulieata breed in Southern Kazakhstan in comparison with other breeds showed a high occurrence of the kappa-casein gene (κ-Cn, CSN3) in animals with genotypes AB and BB, as well as a more frequent occurrence of the B allele, which is important for cheese making. Phylogenetic analysis showed that animals of the Aulieata breed are closest to the German black-pied cattle and are included in a common cluster with them. Although the black-pied alleles are rare in the Aulieata breed, they are positively correlated with the level of milk yield over the 305-day lactation period (Alentayev, 2010).

## Population genetics analysis of the Kazakh white-headed and Alatau breeds

To carry out this analysis, blood samples of the Alatau breed (40 individuals) were used from Kakpatas LLP in Zhambyl region, 53 blood samples of the Kazakh white-headed breed from the Agro Baltabay peasant farm in Almaty region, 25 hair follicle samples were obtained from the Elimay peasant farm, East Kazakhstan region. Genotyping of DNA samples of the Kazakh white-headed and Alatau breeds was carried out using the BovineSNP50 v.3 array (Illumina, USA) following the manufacturer’s protocol at Miratorg-Genetika LLC. The results of the genotyping of these two breeds of Kazakh selection were combined with genotyping data of Altai cattle and closely related breeds from Russia (Yurchenko et al., 2018a) using the PLINK v. 1.9 program (Purcell et al., 2007). Structural analysis of pooled genotyping data from 389 individuals was performed using the fastSTRUCTURE program (Raj et al., 2014).

**Fig. 3.**
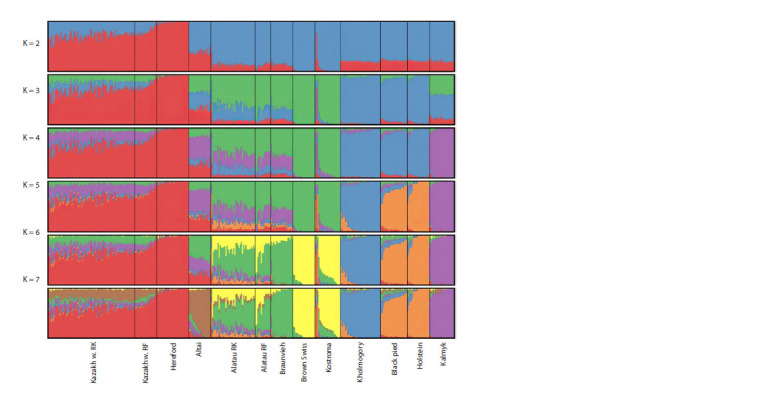
Genetic structure of cattle breeds from Kazakhstan and other Eurasian breeds. Kazakh w. RK – Kazakh white-headed breed from the Republic of Kazakhstan; Kazakh w. RF – Kazakh white-headed breed from the Russian Federation; Altai – Altai cattle; Alatau RK – Alatau breed from the Republic of Kazakhstan; Alatau RF – Alatau breed from the Russian Federation. The results of population clustering using the fastSTRUCTURE program from K = 2 to K = 7 are shown.

Analysis of the genetic structure (Fig. 3) of the populations of the Kazakh white-headed and Alatau cattle breeds of Kazakhstan selection in the context of these breeds from Russia and related breeds show the division of breed groups into two main populations at K = 2.

The first group includes the Hereford breed, and the second group includes the Brown Swiss breed. The remaining breeds have either predominantly Hereford components (Kazakh white-headed of Russian and Kazakhstan selections), or predominantly Brown Swiss components (Altai cattle, Kholmogory, Black pied, Holstein, Kalmyk, Alatau of Kazakhstan and Russian selections, Braunvieh, Kostroma).

At K = 3, a component of the Brown Swiss and Kostroma breeds appears, which distinguishes a group of similar breeds (Brown Swiss, Kostroma, Braunvieh, and Alatau breeds of Kazakhstan and Russian selections). A cluster of dairy breeds becomes clear: Kholmogory, Black pied and Holstein. Altai cattle and Kalmyk breeds appear to be hybrid populations. At K = 4, the Kalmyk breed forms a separate cluster, its unique component can be traced in the Kazakh white-headed breed, Altai cattle, as well as the Alatau breed and Braunvieh. At K > 5 this component disappears in the Braunvieh breed. At K = 5, the Kholmogory breed is separated from the general cluster with Holsteins and the Black pied breed. At K = 6, the structure of the Alatau breed appears, which is composite and has components of the Brown Swiss (Kostroma), Braunvieh, Kalmyk, and Holstein (Black pied) breeds.

The proximity to the Kostroma and Swiss breeds is likely explained by the origin of the Alatau breed. Animals of the Alatau breed of Kazakhstan selection have a slightly more pronounced component of Holstein cattle and the Kalmyk breed compared to the Russian population. The number of animals of Kazakhstan selection of this breed has increased from ~500 heads to 7 thousand over the past 10 years. Thus, the observed differences may be explained by bottleneck effects and genetic drift. At K = 7, Altai cattle form a separate cluster, a component of which is present in the Kazakh white-headed breed. Thus, the Kazakh white-headed breed has a pronounced component of the Hereford breed, Kalmyk and Altai cattle. Altai cattle are probably close in genetics to the original Kazakh cattle used to produce the Kazakh white-headed breed. On average, the Kazakh white-headed breed of Kazakhstan selection has a smaller component of Hereford and a larger component of Altai and Kalmyk cattle compared to the Kazakh white-headed breed of Russian selection

In the last decade, active work has been carried out in Kazakhstan to preserve local livestock breeds, including the Kazakh white-headed breed (www.aqbas.kz). One of the goals of this program is to gradually reduce the use of imported breeds in the breeding of local ones. It is possible that this strategy reduced the fraction of Hereford genetics in the Kazakh white-headed population bred in Kazakhstan compared to the population from Russia. Overall, analysis of the genetic structure of these cattle breeds highlights the importance of conserving and maintaining their genetic diversity to ensure resilience and adaptability to changing environmental conditions and livestock production needs.

## Conclusion

Throughout the long history of livestock farming in Kazakhstan, unique breeds have been developed and adapted to its climatic and environmental conditions, which play a crucial role in the country’s livestock sector. Molecular genetic studies show their closeness not only to European breeds but also to the group of Turano-Mongolian breeds. Recent work on DNA fingerprinting, microsatellites and SNP markers shows that Kazakhstan’s cattle need to be studied in more detail to identify adaptive and unique genetic markers for economically important traits of local breeds. The most promising approach may be whole-genome sequencing of the main cattle breeds of Kazakhstan and their comparison with the genomes of breeds from around the world. The emphasis on preserving the genetic diversity of Kazakhstan’s cattle breeds is consistent with global efforts to maintain the biodiversity of local domestic animal populations.

## Conflict of interest

The authors declare no conflict of interest.
